# Uncertainty analysis of MR-PET image registration for precision neuro-PET imaging

**DOI:** 10.1016/j.neuroimage.2021.117821

**Published:** 2021-05-15

**Authors:** Pawel J. Markiewicz, Julian C. Matthews, John Ashburner, David M. Cash, David L. Thomas, Enrico De Vita, Anna Barnes, M. Jorge Cardoso, Marc Modat, Richard Brown, Kris Thielemans, Casper da Costa-Luis, Isadora Lopes Alves, Juan Domingo Gispert, Mark E. Schmidt, Paul Marsden, Alexander Hammers, Sebastien Ourselin, Frederik Barkhof

**Affiliations:** a Centre for Medical Image Computing; Department of Medical Physics and Biomedical Engineering, University College London Gower Street WC1E 6BT, London, UK; b Division of Neuroscience & Experimental Psychology, University of Manchester, UK; c Wellcome Centre for Human Neuroimaging, Queen Square Institute of Neurology, University College London, UK; d Dementia Research Centre, Queen Square Institute of Neurology, University College London, UK; e School of Biomedical Engineering and Imaging Sciences, King’s College London, UK; f Institute of Nuclear Medicine, University College London, London, UK; g Amsterdam UMC, Vrije Universiteit Amsterdam, Department of Radiology and Nuclear Medicine, Amsterdam, Netherlands; h Barcelonaßeta Brain Research Center (BBRC), Pasqual Maragall Foundation, Barcelona, Spain; IMIM (Hospital del Mar Medical Research Institute), Barcelona, Spain; Centro de Investigación Biomédica en Red de Bioingeniería, Biomateriales y Nanomedicina (CIBER-BBN), Madrid, Spain; i Janssen Pharmaceutica NV, Beerse, Belgium

**Keywords:** PET, MR, Registration, Precision, Partial volume correction, Amyloid, Alzheimer’ disease

## Abstract

Accurate regional brain quantitative PET measurements, particularly when using partial volume correction, rely on robust image registration between PET and MR images. We argue here that the precision, and hence the uncertainty, of MR-PET image registration is mainly driven by the registration implementation and the quality of PET images due to their lower resolution and higher noise compared to the structural MR images. We propose a dedicated uncertainty analysis for quantifying the precision of MR-PET registration, centred around the bootstrap resampling of PET list-mode events to generate multiple PET image realisations with different noise (count) levels. The effects of PET image reconstruction parameters, such as the use of attenuation and scatter corrections and different number of iterations, on the precision and accuracy of MR-PET registration were investigated. In addition, the performance of four software packages with their default settings for rigid inter-modality image registration were considered: NiftyReg, Vinci, FSL and SPM. Four distinct PET image distributions made of two early time frames (similar to cortical FDG) and two late frames using two amyloid PET dynamic acquisitions of one amyloid positive and one amyloid negative participants were investigated.

For the investigated four PET frames, the biggest impact on the uncertainty was observed between registration software packages (up to 10-fold difference in precision) followed by the reconstruction parameters. On average, the lowest uncertainty for different PET frames and brain regions was observed with SPM and two iterations of fully quantitative image reconstruction. The observed uncertainty for the varying PET count-level (from 5% to 60%) was slightly lower than for the reconstruction parameters. We also observed that the registration uncertainty in quantitative PET analysis depends on amyloid status of the considered PET frames, with increased uncertainty (up to three times) when using post-reconstruction partial volume correction. This analysis is applicable for PET data obtained from either PET/MR or PET/CT scanners.

## Highlights

• Novel methodology and software for MR-PET registration uncertainty analysis.
• Registration software had the biggest effect on MR-PET registration precision, followed by reconstruction parameters (i.e., iterations, smoothing) and PET count level.
• PVC can significantly improve the PET signal, but since it relies on precise MR-PET registration, it also increases PET signal variability and hence care should be taken when using it.

## 1 Introduction

*In vivo* quantification is one of the key features of imaging with positron emission tomography (PET), allowing accurate estimation of parameters describing the function and physiology of different organs. However, the quality and robustness of the image derived parameters is highly dependent on a number of factors including physics-, patient-, and reconstruction-specific factors Frey et al. (2012). Among these, the leading physical aspects limiting the precision of PET are the radiotracer together with image noise and limited spatial resolution (also referred to as partial volume effects—PVEs) Barrett et al. (1994); Erlandsson et al. (2012); Hooker and Carson (2019). Furthermore, neuro-PET imaging nearly always requires the use of precisely aligned T1 weighted (T1w) MR image data to provide the necessary soft tissue contrast for the analysis and interpretation of the PET data Schwarz et al. (2017). However, the spatial distribution of various neuro PET radiotracers—combined with the PET noise and limited resolution—can also negatively affect the precision of the MR-to-PET (MR-PET) rigid-body registration and consequently further limit the precision of PET quantification^2^ Therefore, it is important to determine the uncertainty of MR-PET registration and how this propagates to the accuracy and robustness of the quantitative parameters.

**Fig. 1:**
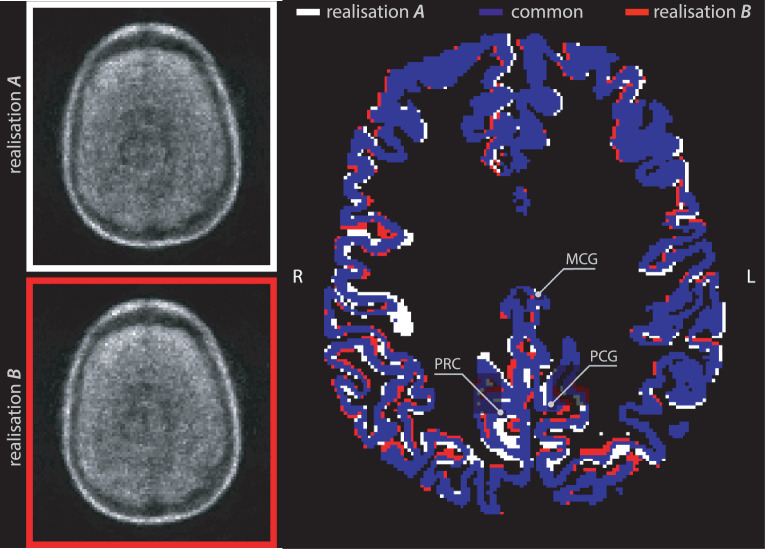
Transaxial illustration of the misalignment imprecision caused by T1w rigid registration to two noisy PET realisations (*A* and *B*) of the same scan shown on the left. The segmented neocortex was propagated to the two PET images using two corresponding rigid-body transformations. The common area of the grey matter to both registrations is shown in blue; the voxel deviations from the common area (the sources of PET quantification error) are shown in white and red. Indicated brain regions: **MCG**: the middle cingulate gyrus; **PCG**: the posterior cingulate gyrus; **PRC**: the precuneus. (For interpretation of the references to colour in this figure legend, the reader is referred to the web version of this article.)

The effect of MR-PET registration uncertainty is illustrated in Fig. 1, where the same brain with two noisy PET image realisations is considered, *A* and *B*. Two independent MR-PET registrations of the T1w image to both PET images were performed, and the transformations were then used to propagate the T1w-based structural segmentations into the PET images. Despite the considerable overlap of the cortical regions shown in blue, there is a significant number of voxels that do not overlap (shown in white and red for realisations *A* and *B*, respectively).

Several methods for estimating PET image noise (and hence uncertainty) have been proposed, such as the interval-based image reconstruction Kucharczak et al. (2018), Bayesian estimation Sitek (2012), approximate variance estimation of regularised reconstruction methods Fessler (1996); Jinyi Qi and Leahy (2000) and variance estimation of the expectation-maximisation (EM) algorithm used in PET image reconstruction Barrett et al. (1994); Wilson et al. (1994). However, the problem with analytically derived approximations of the uncertainty is that it is difficult to further propagate the uncertainty into post-reconstruction processes (e.g., partial volume correction), which generate the final image-based measurements of clinical significance. Nevertheless, other proposed approaches that do not have this limitation also exist, which are based on bootstrap resampling of raw PET data Buvat (2002); Lartizien et al. (2010); Markiewicz et al. (2015). The advantage of resampling methods is that they can account for the whole image generation process, including all reconstruction algorithms with their specific corrections for scatter and random events, and particularly, can propagate the uncertainty through any additional processes and analyses. On the other hand, the disadvantage of resampling methods is their high computational cost, requiring longer times for the uncertainty estimation.

The novelty of this work lies in the uncertainty analysis, which helps identify the image processing aspects that need optimisation for more precise estimation of subtle changes in PET signal, e.g., in longitudinal measurements of amyloid deposition in the brain. This analysis focuses on comprehensive investigation of the impact of PET image parameters on the precision of MR-PET image registration and its impact on the final PET image statistics. This analysis is enabled by new methodological and software aspects based on GPU rapid generation of bootstrap realisations to determine the voxel-level PET noise Markiewicz et al. (2016). The uncertainty of MR-PET registration is assessed using four distinct PET radio-distributions, while systematically varying (1) the image reconstruction parameters, such as the number of iterations and selection of attenuation and scatter corrections; (2) the resampled PET count level relative to the chosen gold standard and (3) registration software packages. Such an analysis will enable more informed choices of image processing for high precision quantitative PET analysis. Although this work focuses on the most common PET reconstruction parameters, the assessment of future and more advanced reconstruction methods together with other PET radiotracers will be added on a regular basis on our website https://niftypet.readthedocs.io.

## 2 Methods

### 2.1 PET/MR Data acquisition and processing

The participants for this investigation came from the Insight’46 cohort study—a neuroscience sub-study of the UK’s Medical Research Council National Survey of Health and Development Lane et al. (2017). The participants used in this study were two cognitively normal females: one amyloid negative (69 years old at the time of scan) and one amyloid positive (71 years old). All PET and MR data were obtained from a simultaneous PET/MR scanner—the Siemens Biograph mMR. Data processing, such as PET list-mode resampling, quantitative corrections for photon attenuation, scatter and randoms, followed by image reconstruction and post-processing were performed using the high-throughput Python package *NiftyPET* Markiewicz et al. (2018) (https://niftypet.readthedocs.io).

The core of the uncertainty analysis was performed using four distinct radio-distributions obtained from two dynamic amyloid PET scans ([18F]florbetapir) acquired in list-mode for 60 minutes—one was clearly positive and the other clearly negative for amyloid. To limit the confounding effects of motion, both scans were chosen to have no, or minimal, head motion. PET image reconstruction was performed using the ordered subsets expectation maximization (OSEM) algorithm Hudson and Larkin (1994) with varying number of iterations and different quantitative correction setups. The PET reconstruction for MR-PET registration was considered as separate from the standard quantitative PET reconstruction—for example, in case of correcting for motion, the PET may be reconstructed without attenuation correction in order to register the μ-map to the PET image, after which a correct attenuation correction may be applied. Also, for some PET distributions, the non-attenuation corrected images yield better registrations Costes et al. (2009). Corrections for attenuation and scatter were by default performed using the attenuation maps based on ultrashort echo time (UTE) MRI sequences aligned to PET. However, since attenuation correction with PET/MR is still challenging, for comparison purposes and comparability with PET/CT, μ-maps based on synthetic CT images (referred to as pseudo-CT, pCT) were generated and also used for attenuation correction Burgos et al. (2015). The registrations of UTE and T1w MR images to PET for μ-map alignment were performed independently from the MR-PET registrations in the uncertainty analysis. Note, that the reconstruction for registration is performed separately from the standard PET reconstruction, focusing on their suitability for MR-PET registration rather than PET quantification.

Structural parcellations of the brain based on the T1w image were used for regional quantitative PET analysis. The analysis was performed in the native PET space to avoid any quantification errors due to PET image transformations and interpolation. The parcellations were obtained using the geodesic information flow (GIF)—a multi-atlas segmentation propagation strategy Cardoso et al. (2015). The position of the T1w image and the corresponding parcellations were randomly perturbed (simulating the random relative position of PET and MR image pairs) using the NIfTI affine matrix of the T1w and parcellation images. After image registration, the parcellations were propagated to the native PET space using the rigid body transformation and nearest neighbour interpolation.

### 2.2 Uncertainty analysis

**Fig. 2:**
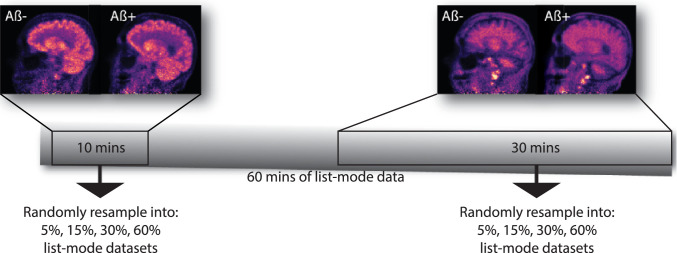
The selection of two time frames from the dynamic PET acquisition used for random resampling of the PET data for uncertainty analysis of MR-PET registration due to different PET noise levels. Note the distinctly different patterns of white/grey matter distributions of the late 30-minute frames—greater uptake in the grey matter in the amyloid positive case with lost contrast between the grey and white matter.

**Fig. 3:**
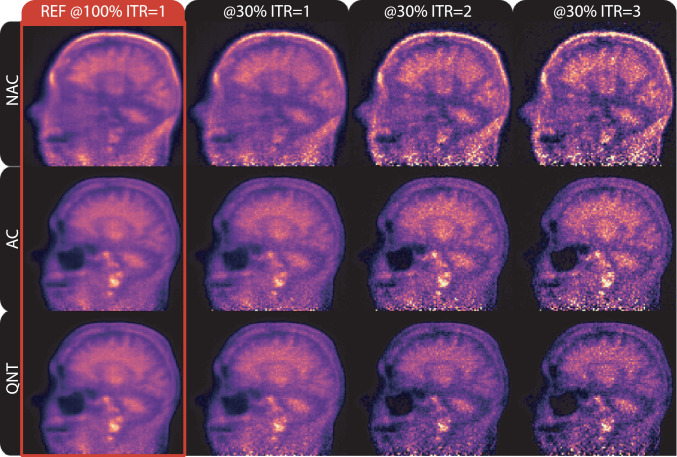
Variable reconstruction parameters for the uncertainty analysis. Shown is the negative Aβ late frame scan, reconstructed at full counts for the reference, and at 30% count-level with 1, 2 and 3 OSEM iterations (ITR). The three reconstruction setups are: without attenuation correction (NAC), with attenuation correction (AC), and fully quantitative (QNT).

The following variables were investigated in the MR-PET registration uncertainty analysis:

(A) Temporal radio-tracer distribution as in early and late dynamic frames.
(B) PET reconstruction parameters, i.e., the number of iterations and application of attenuation and scatter corrections (on/off).
(C) Frame count level at 5%, 15%, 30% and 60% relative to the full count frame, using PET list-mode data resampling.
(D) Rigid body image registration software packages.
(E) PET voxel size.
(F) Initial position of the MR image relative to the PET image.

(A) *Early and late PET radio-tracer distributions.* Two distinct PET tracer distributions for both dynamic scans were considered: the early and late time frames as shown in Fig. 2. The radio-distribution of the first time frame of 10 minutes has been shown to be very similar to [18F]fludeoxyglucose (FDG) and provides information about cerebral blood flow Hsiao et al. (2012). The late frame acquisitions for the amyloid tracer are markedly different from the early distributions and provide information about amyloid deposition in grey matter of the brain. For good count statistics, the last 30 minutes of both PET list-mode acquisitions were used for the late frames.

(B) *Variable PET reconstruction parameters.* Three image reconstruction setups were investigated in the uncertainty analysis: (i) without attenuation correction (NAC), (ii) with attenuation correction (AC); and (iii) fully quantitative reconstruction (QNT), adding scatter correction to (ii). Corrections for random events, dead-time and detector normalisation were performed in all setups. In addition, one to three OSEM iterations with 14 subsets were used as early stopping of iteration as means of controlling the PET image noise Tong et al. (2010). All images were generated by bootstrap resampling at 30% count-level relative to the full-count reference image. Fig 3 shows the three reconstruction setups and three iterations for the late frame of the amyloid negative scan. The different reconstruction parameters were used only for the purpose of MR-PET registration and not for PET quantification, which is based on a separate PET reconstruction. The uncertainty analysis for the reconstruction parameters was based on 600 independently resampled PET list-mode datasets (4 frames × 3 iterations × 50 bootstraps), which were reconstructed with three different numbers of OSEM iterations, resulting in 1800 PET images. An additional 1200 PET reconstructions were performed for aligning the μ-map for AC and QNT reconstruction modes, making up a total of 3000 PET images used in this analysis.

(C) *Variable count-level PET data resampling.*

The noise level of the T1w images is usually significantly lower than that of PET images, which are based on limited count statistics in short time frames to capture the PET tracer kinetics and head motion, while keeping the radiation dose as low as possible. Therefore, it is likely that the uncertainty of MR-PET registration is considerably affected by the PET noise. Multiple variable count-level PET images were generated using the bootstrap, which simulates a new ‘measurement’ from the probability distribution represented by the original measurement Herholz et al. (2014); Markiewicz, Reader, Matthews, 2015, Markiewicz, Thielemans, Schott, Atkinson, Arridge, Hutton, Ourselin, 2016. The duration of the PET frames were chosen to be long enough (Fig. 2) to ensure good statistics of the reference PET image, resulting in a corresponding reference MR-PET registration (treated as the gold standard here), to which registrations based on lower count PET images are compared.

**Fig. 4:**
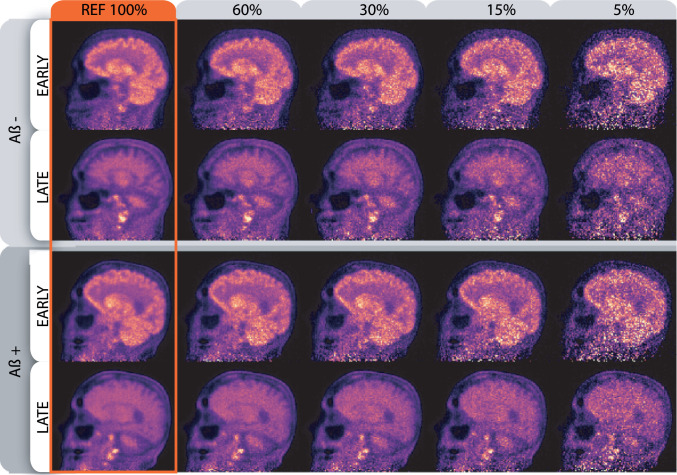
Variable count-level for the uncertainty analysis. In rows are shown negative and positive amyloid scans (2 iterations of AC OSEM), each with an early time frame (80–680 s) and a late time frame (1800–3600 s). The variable count level is shown in columns, varying from 60% to 5%, with the full count-level acting as a gold standard and reference (most left column).

The chosen count-levels were 60%, 30%, 15% and 5% of the full frame. The variable count-level PET images for the amyloid positive and negative scans, as well as for the early and late frames, are shown in Fig. 4. The typical count-level of a clinical static amyloid scan is around 30% of the 30 minute frame. For each count-level (4), 50 bootstrap realisations of raw PET data were generated and independently reconstructed, which, together with the reference (full-count) PET image, were used for estimation of the distribution of the MR-PET registration imprecision. Thus, 800 independent bootstrap list-mode datasests were generated and reconstructed using AC OSEM, with another 800 reconstructions used for aligning the UTE-based μ-map for attenuation correction. In addition, two PET image voxel sizes (2.0 and  1.0 mm isotropic) were investigated, both used in registration with and without perturbations of the MR relative position. A total of 2400 PET images were generated for this.

(D) *Different software packages for MR-PET registration:* For each MR-PET image registration, four different registration software packages were used to investigate uncertainties introduced by different software implementations. The software packages were: (**i**) Statistical parametric mapping (SPM) 12 with update revision number 7487 (see www.fil.ion.ucl.ac.uk/spm/); (**ii**) NiftyReg (ver. 1.5.61)—a global registration using a symmetric block-matching approach Modat et al. (2014) (https://github.com/KCL-BMEIS/niftyreg/wiki); (**iii**) VINCI (Volume Imaging in Neurological Research, Co-Registration and ROIs included) version 4.95 (http://vinci.sf.mpg.de/); (**iv**) the FMRIB’s Linear Image Registration Tool (FLIRT) from FSL package version 6.0 (https://fsl.fmrib.ox.ac.uk/fsl). However, since FSL has not been optimised to work with PET, the registrations were failing due to lack of brain extraction, which is not trivial in PET, and on which FSL relies for precise registration. For all the registrations, the cost function of normalised mutual information was used, apart from NiftyReg which was based on a block-matching technique and least-trimmed square regression. The default (off-the-shelf) settings were used for all the packages. Note also, this investigation is not aiming to compare the software packages, which can always be modified and improved for any given task, but rather to show how different software can impact PET analysis.

(E) *Image pre-processing.* The T1w images with voxel size of 1.1 mm isotropic have significantly higher resolution compared to the native PET with voxel size of 2.09 × 2.09 × 2.03 mm${}^{3}$. Therefore, the PET images were upsampled by dividing each voxel into eight equal voxels (without interpolation), resulting in a 1.04 × 1.04 × 1.01 mm${}^{3}$ voxel size, and thus enabling the use of high resolution ROI definitions based on the T1w images. All T1w MR images were corrected for geometric distortions and the bias field.

(F) *Perturbation of the MR image position:* Since the data under investigation comes from a simultaneous PET/MR scanner, it is likely that the T1w image will be in close register with the PET image. This would be, however, unlikely to be the case when PET and MR scans are acquired at different times. Therefore, for each PET bootstrap realisation, the position of the MR image was randomly perturbed by modifying the NIfTI image affine matrix, and thus resulting in unique spatial position of all MR-PET pairs to be registered. Similarly to Schwarz et al. (2017), the perturbations consisted of added random translations in each direction (x,y,z) of between -10 and 10 mm, as well as random rotations between $-10^{\circ }$ to $+10^{\circ }$ around each axis, leaving the voxel values intact.

### 2.3 Quantification of registration uncertainty.

The key element of the uncertainty analysis is the quantification of the MR-PET registration precision. This was performed by using two metrics: (1) the standardised uptake value ratio (SUVr)—a ratio between target and reference regions; (2) the Dice coefficient of the registration transformations relative to the gold standard transformation based on high statistic PET. The first metric is used on a single, typical clinical quantitative PET scan, which was acquired for 10 minutes (50 minutes post injection–the last 10 minutes of the list mode data), and was sampled using ROIs of variable position subject to the MR-PET registration imprecision as estimated by the resampled PET images used in the uncertainty analysis above. Thus the observed uncertainty came from registration imprecision only, while the effects of noise of the target quantitative PET itself were not considered. In addition, the effect of partial volume correction (PVC) was also investigated, by using the iterative Yang algorithm on the target quantitative PET with varying definitions of the ROIs for each bootstrap realisation Erlandsson et al. (2012); Markiewicz et al. (2018). The PVC correction was performed post image reconstruction and required already aligned brain parcellations as input.

In addition, we investigated the rigid-body transformations themselves, with suitable metrics being the Dice coefficient and Jaccard index Taha and Hanbury (2015), both of which require the T1w parcellations propagated to the corresponding PET spaces. Although both metrics are closely related, the more frequently used Dice coefficient was selected Dice (1945). The Dice coefficient for a single pair of the reference and bootstrap parcellation images, $S_{r}$ and $S_{b},$ respectively, is defined as:

(1) $$DICE=\frac{2\left|S_{r}\cap S_{b}\right|}{\left|S_{r}\right|+\left|S_{b}\right|}.$$

**Fig. 5:**
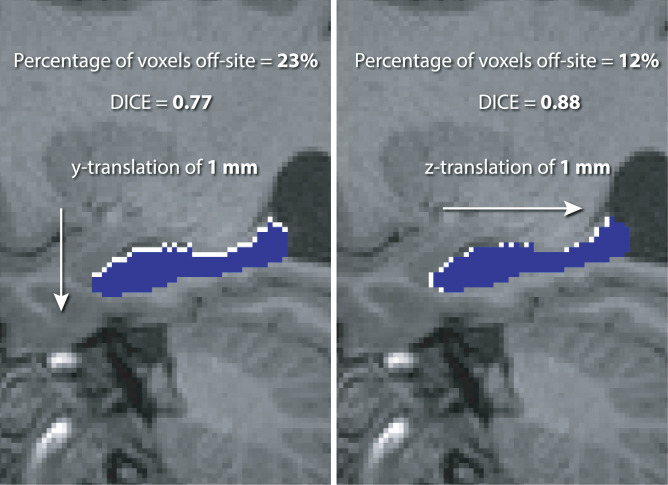
The reason for using the Dice coefficient as a direct overlap metric of the precision of MR-PET image registration. Metrics based on translations, and similarly rotations, are not accurate predictors of ROI sampling precision as shown for the hippocampus (represented by the white ROI in the background) and two equally misplaced ROIs (represented by the blue ROI in the foreground) along *y* and *z* axes by 1 mm each, respectively, producing significantly different sampling errors (23% vs. 12% error for y-translation vs. z-translation). (For interpretation of the references to colour in this figure legend, the reader is referred to the web version of this article.)

The registration uncertainty for any given ROI is estimated by forming a distribution of the Dice coefficients of all 50 bootstrap realisations relative to the single reference parcellation. The advantage of the Dice coefficient is that it quantifies effects of misalignments in rotations and translations more accurately than quantifying the rotations or translations separately as is illustrated in Fig. 5 where two 1-mm translations produce different ROI sampling errors.

## 3 Results

**Fig. 6:**
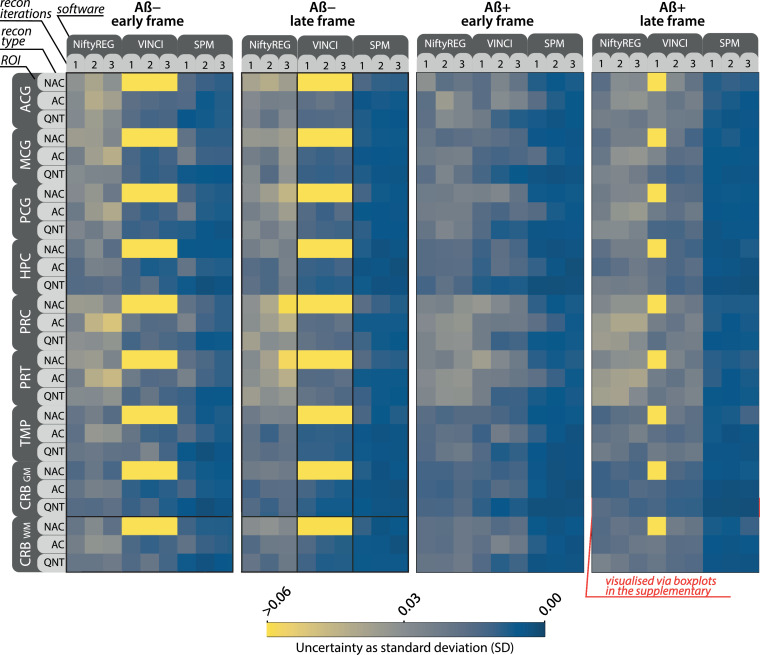
Matrices of standard deviations (SD) of the Dice coefficient as a measure of MR-PET registration uncertainty across 9 grey-matter-only ROIs shown in 9 block rows, which are: the anterior, middle and posterior cingulate gyri (ACG, MCG and PCG, respectively), the hippocampus (HPC), precuneus (PRC), parietal (PRT) and temporal (TMP) lobes, and the cerebellar grey (CRB GM) and white (CRB WM) matter. Investigated are early and late frames of amyloid negative (Aβ−) and positive (Aβ+) PET scans shown in the four groups from left to right. The uncertainties for reconstruction parameters are shown as 3×3 pixel matrices composed of one to three OSEM iterations (columns) and three quantification corrections modes (rows): with no attenuation correction (NAC), with attenuation correction (AC), and fully quantitative (QNT). PET count-level was at 30% compared to the reference 100% for each Dice coefficient measurement. The white dot indicates lowest recorded uncertainty. The SDs marked with the red box are shown as boxplots in Fig. 11 in the supplementary material. (For interpretation of the references to colour in this figure legend, the reader is referred to the web version of this article.)

A total of 5400 PET images were generated, for which MR-PET registrations were performed using four different software implementations, resulting in 16,800 registrations (for the count-level analysis the registration was performed with and without MR position perturbation). The MR-PET registration uncertainty is presented across nine ROIs in Fig. 6, using the metric of standard deviation (SD) of the distributions of the Dice coefficient for all reconstruction setups, the four registration software packages and the four PET frames. Each SD pixel in Fig. 6 is derived from 50 reference-bootstrap Dice coefficients. The most precise registrations were obtained using SPM for the cerebellum grey matter, apart from the late frame of amyloid negative scan, for which it was obtained for the hippocampus. The observed difference in precision between software implementations was up to 10-fold, which was observed for the temporal lobe of the early frame of negative amyloid scan reconstructed with QNT OSEM and two iterations. For the AC OSEM reconstruction, the observed difference in precision was 9-fold obtained for the hippocampus and the negative amyloid late frame reconstructed with three OSEM iterations. The boxplots of the distributions marked by the red box are shown in the supplementary material such that the precision (shown in the width of the boxplots) can be compared to the obtained accuracy (closeness to the value of 1) for the different registration software packages (Fig. 11 in the suplementary).

**Fig. 7:**
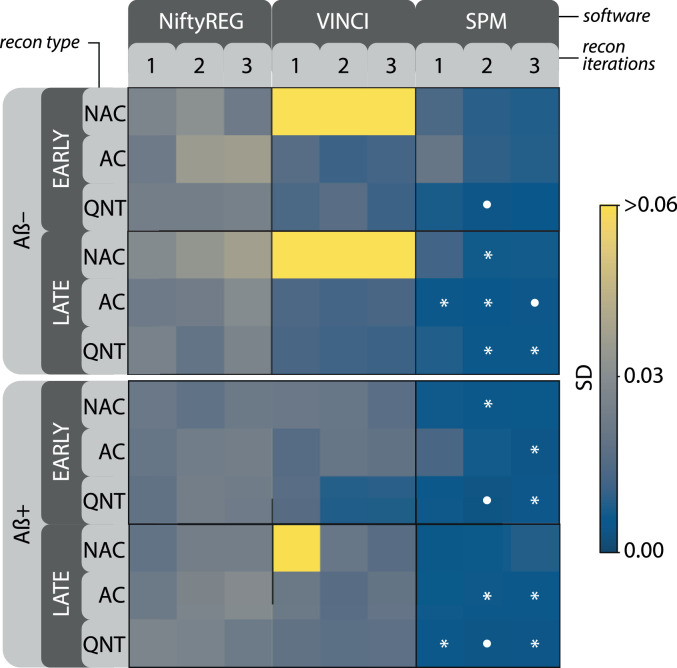
Matrices of **average** standard deviations (SD) of the Dice coefficient across 9 different brain regions of interest (ROI)—a reduced version of Fig. 6—as a measure of average MR-PET registration performance for different PET distributions (early and late frames and amyloid positive and negative scans), PET reconstruction types, and image registration software packages. Count level was at 30%. The white solid dot indicates lowest recorded uncertainty for each frame while the asterisks indicates uncertainties which are statistically indifferent to this uncertainty.

The average performance across the nine ROIs is shown in Fig. 7. The best average registration performance was obtained with SPM registration, the quantitative PET reconstruction, two OSEM iterations, apart from the late frame of amyloid negative scan, for which the best results were obtained using AC reconstruction with three iterations. The parameters yielding best precision were marked with solid dot, while the parameters yielding statistically indistinguishable precision as tested with the Brown-Forsythe test for equal variances were marked with the asterisk. The bright-yellow SD values correspond to distributions in which at least one registration failed, thus significantly increasing the SD values.

The PET reconstruction parameters can also have a significant effect, for example, the highest observed average range of SD values due to reconstruction parameters across all nine ROIs using SPM was 0.016 (4-fold change) for the early frame of negative amyloid scan (minimum SD = 0.0043 for QNT reconstruction with two iterations of OSEM and maximum SD = 0.02 for AC reconstruction with one iteration).

**Fig. 8:**
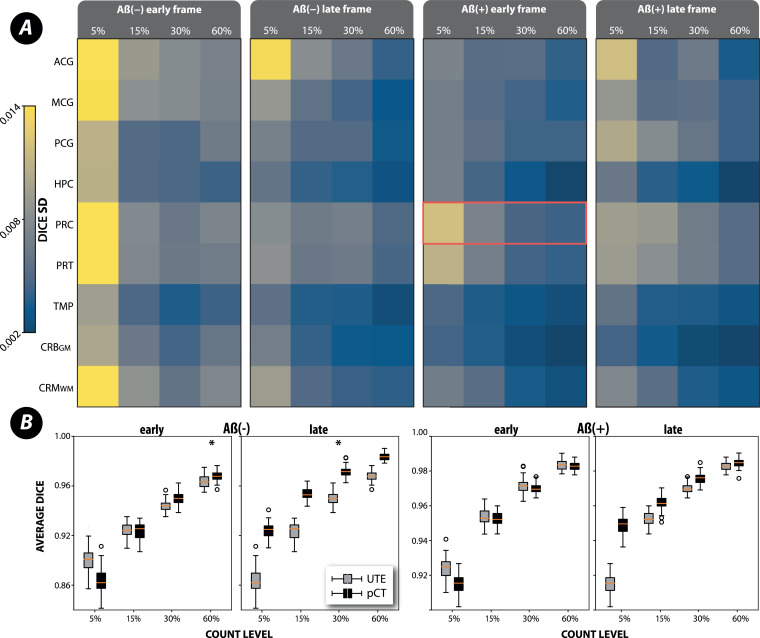
The SPM12 registration uncertainties for different PET count levels and PET distributions. **A:** The uncertainties are presented as standard deviation for multiple regions of interest (ROI) of early and late frames of amyloid positive (Aβ+) and negative (Aβ−) PET scans. The red box marks the largest peak-to-peak uncertainty between the count-levels (cf. Fig. 12 in the suplementary). **B:** The average performance across all ROIs is shown as boxplots using two iterations of AC OSEM with UTE (grey) and pCT (black) μ-maps. (For interpretation of the references to colour in this figure legend, the reader is referred to the web version of this article.)

The precision of MR-PET registration for four count-levels, four PET distributions and the nine ROIs using SPM12 for registration is shown in Fig. 8 using standard deviation (SD) and boxplots. Fig. 8*A* shows the SD of the Dice coefficient distribution across all ROIs for PET images reconstructed with UTE μ-map. Fig. 8*B* shows the pooled Dice coefficient distributions across the ROIs and PET images reconstructed with the UTE and pCT μ-maps. The marked red box for the precuneus ROI of the early frame of the Aβ+ scan exhibits one of the highest uncertainty ranges across the count-levels and is further investigated together with the simulation of random relative position of PET and MR images (see the supplementary material).

**Fig. 9:**
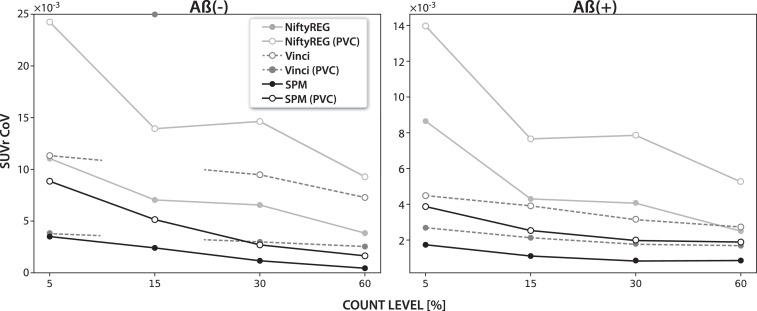
The effects of MR-PET registration uncertainties on the precision of the SUVr of the precuneus ROI, reported as the coefficient of variation for four different count-levels of negative (left) and positive (right) amyloid PET scans. The cerebellum grey matter was used as the reference ROI. Standard and partial volume corrected (PVC) PET images were considered for the calculation of the SUVr metric..

The uncertainty of the PET imaging endpoint SUVr due to the MR-PET registration imprecision is shown in Fig. 9 for the two late PET frames with and without partial volume correction (PVC) and for the three registration software packages. The precuneus was considered as the target region and cerebellum grey matter as the reference region. The uncertainty is reported as the coefficient of variation (CoV) and represents only the loss of precision due to the MR-PET registration and not the variability of the PET signal (which is yet another source of variability). Note that for the Vinci software at 15% count level the point is not included as for one noise PET realisation the registration failed, significantly increasing the variation beyond the scale. The boxplots representing the distrubiton of Dice coefficient for SPM have been added in the supplementary material.

## 4 Discussion

The uncertainty of MR-PET registration was quantified and evaluated using high-throughput dedicated imaging pipelines for resampled datasets. The effects of PET distribution, count-level and image reconstruction parameters on MR-PET registration were assessed using four different registration software packages.

*The choice of registration software* had the biggest impact on the registration uncertainty with SPM12 achieving highest precision and accuracy. Note that the range of the standard deviation of the Dice coefficient observed for the different registration packages in Fig. 6 and 7 is around 5 times bigger than that of the count-level as shown in Fig. 8. Note that the investigation of different registration parameters for each software package was beyond this work. Instead, we focused on the default, off-the-shelf parameters as they are commonly used. Therefore, these results cannot be used to unequivocally indicate which software is more precise, but merely to quantify the impact of using different software packages. Since SPM12 with the default settings achieved highest precision, it was used for the uncertainty analysis using variable PET count-levels as shown in Fig. 8. Although this work has been limited to the few registration software packages, it will be gradually expanded including other registration packages (such as FreeSurfer, ANTs, DIPY) and published online on our website https://niftypet.readthedocs.io/.

*The average registration performance* across ROIs may be useful to know when performing multi-regional brain analyses. The optimal registration was achieved with two iterations of OSEM and full quantitative PET reconstruction, with the exception of late frame of Aβ(−), for which three OSEM iterations with attenuation correction only was slightly better (Fig. 7). Hence, this AC reconstruction with two iterations may be further investigated across multiple subjects for detection of subtle and early changes of amyloid deposition as it also allows faster processing without performing scatter correction.

*The reconstruction parameters*, such as the number of OSEM iterations and the quantification corrections, had the second highest impact on precision (when statistically significant, see Fig. 7). For example, one OSEM iteration may not be enough for the PET images to produce the highest precision in many SPM registrations (Fig 6), particularly when using AC reconstruction. On the other hand, for NiftyReg, the best average performance was observed with one OSEM iteration in most cases, which would suggest that NiftyReg favours smoother PET images, which can be obtained with fewer number of OSEM iteration. The cerebellum, which often acts as a reference region, was observed to exhibit one of the most precise MR-PET registrations across the considered PET frames. Since the number of OSEM iterations depends on the implementation of the reconstruction and the scanner itself, such an uncertainty analysis should ideally be run on a pilot study to discover the optimal image processing parameters for large studies.

Note, that due to the frequent mismatch between attenuation and emission data caused by motion, registration to PET images without attenuation and scatter corrections may be more accurate, which can be followed by another registration using AC or QNT PET images based on the aligned μ-map for better precision. Furthermore, the optimal registration will depend on the radiotracer distribution and the application—e.g., for some neuroreceptor studies the NAC reconstruction were reported to be optimal Costes et al. (2009); Reilhac et al. (2018). The current scope of this work has been limited to default and easily available reconstruction methods. However, more advanced reconstruction and processing methods will be added to our regularly updated website. The additional investigation will include resolution modelling, PVC with the scanner specific PSF, and dedicated reconstruction with priors which may also help further reduce the uncertainty of MR-PET registration.

*PET count-level* had the third highest impact on the precision of MR-PET registration within the range of count-levels considered, i.e., from 5% to 60% with 30% closely corresponding to the typical 10 minute static acquisition for SUVr quantification. In particular, the results shown in Fig. 8 provide evidence that the PET count-level is strongly and positively associated with the precision, i.e., the higher the count-level the higher the registration precision across all four frames. Importantly, the choice of μ-map (UTE or pCT) overall did not make a statistical difference for the uncertainties using the Brown-Forsythe test (apart from two exceptions marked with asterisks in the boxplots in Fig. 8*B*, p<.05). However, for the late frames, the pCT μ-map produced more accurate (less biased) registrations relative to the reference registrations based on 100% PET count level.

*The randomisation of the relative position of MR to PET* was applied to all the analyses, however, although observing similar registration uncertainties to Schwarz et al. (2017), we found that the random initial position of PET images relative to MR had little effect on the uncertainty compared to the PET count-level (see the supplementary for more details).

*The accuracy* of MR-PET registrations was estimated by comparing the lower-count PET registrations to the full-count PET registration. High precision registration is indicated by the narrow boxplot Dice distributions, and high accuracy is indicated by the distributions being close to the value of 1 (compare Fig. 8*B* as well as Figs. 11 and 12 in the supplementary material). The estimated accuracy (bias) is only approximate as the high-count PET registration is unlikely to be fully representative of the exact and perfect registration which can only be known with infinite PET counts. Nevertheless, all the high precision registrations consistently achieve higher accuracy than the rest.

*The effects of registration uncertainty on PET image analysis* can be particularly seen in quantitative analysis of a single PET image while using variable ROIs definitions due to MR-PET registration imprecision (Fig. 9, see Fig. in the supplementary material for the absolute SUVr values). The presented results are in agreement with Brendel et al. (2015); Gonzalez-Escamilla et al. (2017); Rullmann et al. (2016) where the PVC increased the SUVr value contrast between the positive and negative amyloid cases. This is caused by the SUVr value decrease in the amyloid negative case and SUVr value increase in the amyloid positive case (Fig. 13 in the sumplementary). However, the studies did not investigate the PVC precision. In Frouin et al. (2002), it was shown that the MR-PET registration is a major source of error, decreasing the precision of the PVC. In our study we were able to quantify the loss of precision using real brain scans.

The uncertainty of the SUVr depends on the target ROI and the reference ROI by which the target ROI is normalised. The choice of the registration software had the biggest impact on the precision of the SUVr (more than four times), followed by the partial volume correction, which accounts for the loss of precision of up to 2.5 times in the low-count-based registrations. Also, the effect of PET count-level was greater in the amyloid negative than in the amyloid positive case. Doubling the count-level can reduce the uncertainty by half or more (as measured by the coefficient of variation, CoV). The greater uncertainty in the amyloid negative case is likely due to the greater contrast between cerebral grey and white matter compared to the amyloid positive scan, for which the contrast is lost. However, due to more motion being expected in amyloid positive subjects, the overall precision across these subjects can be lower.

Although the application of PVC to PET can introduce additional uncertainty due to its dependence on the ROI definitions and other factors, the signal increase due to PVC can be greater, as shown by Brendel et al. (2015); Gonzalez-Escamilla et al. (2017); Rullmann et al. (2016). Hence, the ratio of signal to noise may prove to be greater, making the PVC worthwhile. However, it is surmised that the gain in signal can be lower for smaller and narrower ROIs, for which the PET noise and registration uncertainty are greater and therefore care should be taken when applying PVC to PET images Thomas et al. (2011). This additional uncertainty observed with PVC would probably also be observed for any other PVC method, as the source of the uncertainty is the ROI mispositioning caused by MR-PET registration imprecision, regardless of the bias introduced by any PVC method Frouin et al. (2002); Gonzalez-Escamilla et al. (2021); Minhas et al. (2018). Such an insight into the uncertainty of the final image statistic is particularly important for longitudinal imaging, for which the scope of the uncertainty will define the accuracy and speed of early detection of amyloid accumulation or the response to a new therapy. Note, that the choice of the reference region can impact the SUVr precision, as it has been shown with the eroded white matter ROI to increase precision. However, this region in not suitable for dynamic studies and it is unstable across a wider age spectrum Lowe et al. (2018).

*Recommendations for higher precision PET analysis:* In order to ensure the highest possible precision of PET analysis supported by MR-based ROI definitions, we recommend performing uncertainty analysis for any application-specific PET study—the software used in this analysis will be publicly available as open source at https://niftypet.readthedocs.io. Such an analysis may help to choose the optimal registration software and construct PET frames with better noise properties (e.g., by summing or widening the frames when possible). Performing a separate post-processing or PET reconstruction for registration purposes should be considered when the target PET images are too noisy, e.g., in the early dynamic frames, for which longer frames can be considered; or in case of motion correction for which frames are selected in accordance with the occurred motion, more accurately separating different motion zones in the PET acquisition. Choosing custom reconstruction parameters (e.g., greater smoothness obtained with fewer OSEM iterations) may improve the registration. Although, PVC increases the imprecision of the final image statistic (e.g., SUVr) it may still be worthwhile as the gains in PET signal can be greater than the loss of precision.

## 5 Conclusions

Based on the presented uncertainty analysis, we found that the precision of MR-PET image registration depends most strongly on the registration software used and the quality of the PET image as influenced by different reconstruction parameters and the count level. Negative amyloid scans are subject to greater ROI sampling uncertainty due to the higher grey/white matter contrast as opposed to the amyloid positive scans, and hence greater care should be taken when imaging participants at the early stages of amyloid accumulation. Performing PET partial volume correction can introduce additional noise, especially when the MR-PET registration is based on lower quality PET images. This uncertainty analysis opens a way for a development of optimal image reconstruction algorithms with the main emphasis of reducing the image noise while maintaining good contrast for high precision registration. Therefore, it may be beneficial to run a separate PET reconstruction, different from the target PET quantification but dedicated to and optimised for high precision MR-PET registration, which would then facilitating higher precision quantitative PET.

## CRediT authorship contribution statement

**Pawel J. Markiewicz:** Conceptualization, Investigation, Methodology, Software, Formal analysis, Writing - original draft. **Julian C. Matthews:** Formal analysis, Methodology. **John Ashburner:** Methodology, Writing - review & editing. **David M. Cash:** Resources, Data curation, Writing - review & editing. **David L. Thomas:** Resources, Writing - review & editing. **Enrico De Vita:** Resources, Writing - review & editing. **Anna Barnes:** Resources. **M. Jorge Cardoso:** Software. **Marc Modat:** Software. **Richard Brown:** Investigation, Validation. **Kris Thielemans:** Validation, Writing - review & editing. **Casper da Costa-Luis:** Visualization, Software, Writing - review & editing. **Isadora Lopes Alves:** Project administration, Writing - review & editing. **Juan Domingo Gispert:** Validation, Writing - review & editing. **Mark E. Schmidt:** Supervision, Conceptualization. **Paul Marsden:** Supervision, Conceptualization. **Alexander Hammers:** Supervision, Writing - review & editing. **Sebastien Ourselin:** Funding acquisition, Supervision, Conceptualization, Resources. **Frederik Barkhof:** Funding acquisition, Supervision, Conceptualization, Writing - review & editing.

## Appendix A Supplementary materials

Supplementary Data S1 Supplementary Raw Research Data. This is open data under the CC BY license http://creativecommons.org/licenses/by/4.0/ Supplementary Data S1

## Footnotes

2 Even when a PET-MR scanner is used where the data is acquired simultaneously, spatial alignment of the data cannot be assumed due to the sequential nature of MR sequences and subject movement.
