# Supplementary material for: Uncertainty analysis of MR-PET image registration for precision neuro-PET imaging
Source: Neuroimage. 2021 May 15;232:117821. doi: 10.1016/j.neuroimage.2021.117821 (PMC8204268; doi:10.1016/j.neuroimage.2021.117821)
Supplement: Supplementary Data S1 — Supplementary Raw Research Data. This is open data under the CC BY license http://creativecommons.org/licenses/by/4.0/ [file mmc1.pdf]

Supplemental material

PET voxel size in MR-PET registration

The uncertainty analysis was also perfomed with two PET voxel sizes but without the absolute registration gold standard available for both settings, it was not possible to indicate which is more advantageous. The original 2 mm and upsampled (1 mm) PET voxel representations were considered in the analysis as depicted in Fig. 10. Note that usually brain scans are acquired in whole-body PET/CT or PET/MR scanners which have large field of view (FOV) and which is visible in the figure of the original PET image relative to the trimmed and upsampled image.

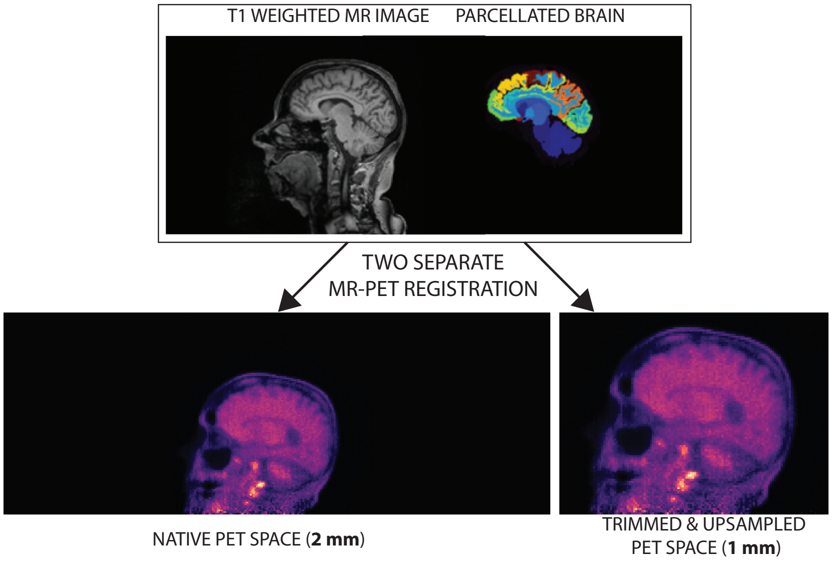

Figure 10: Two separate MR registrations to PET in (1) the original voxel space (default) using a voxel size of 2 mm (**bottom left**) and upsampled and trimmed PET space, with a voxel size of 1 mm (**bottom right**). The brain parcellation (**top left**) is based on the T1 weighted MR image (**top right**), and is resampled to the trimmed PET space using the rigid body transformations.

Boxplots of the Dice distributions for MR-PET registration precision

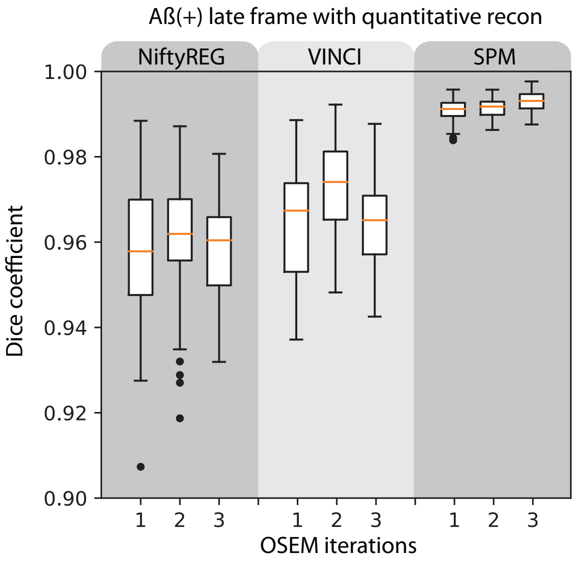

Figure 11: Boxplots of MR-PET registration uncertainties for cerebellum grey matter ROI and late frame of Aβ+ PET scans, which were reconstructed with OSEM and all quantitative corrections. The boxplots are shown for the three different registration software packages.

We used high-count PET images to serve as the gold standard reference against which all the MR-PET registrations test were compared. This is based on the evidence-based assumption that PET images with higher count level yield more precise and accurate registration as further elaborated below. The registration precision and accuracy can be assessed using boxplots as shown in Fig. 11 (the boxplots correspond to the red box marked in Fig. 6). The uncertainty is shown for the cerebellar grey matter and late frame of the Aβ+ scan with fully quantitative reconstruction (QNT) using 30% of the original frame count-level. This count-level is approximately equivalent to the count-level of the last frame of 10 minutes typically used in amyloid imaging. The narrower boxplots indicate higher precision, while the boxplots whose Dice coefficient values are close to 1.0 indicate better association of the bootstrap-based ROI with the gold standard ROI, which is higher accuracy. Not surprisingly, the high pre-

cision distributions also exhibit higher accuracy, which is true for SPM-based registration. Note, that every boxplot of the Dice distribution, as presented for each software and OSEM iteration number, uses the same registration software and number of OSEM iterations for the gold standard parcellation. Hence, each software has its own gold standard to achieve for a fair comparison, with SPM approximating its gold standard more closely than the other registration software packages.

Impact of relative position of MR and PET on the registration precision

The effect of initial position of the MR image relative to the PET has been investigated by randomly perturbing the translations and rotations encoded in the affine matrix of the NIfTI MR image header for each and every bootstrap realisation independently. This change of the initial conditions for the registration algorithm does not introduce any interpolation to either image used in the registration. The effect of the random perturbation on the uncertainty analysis relative to the situation without such a perturbation is shown in Fig. 12 for the early frame of the Aβ+ scan as marked in red in Fig. 8. The distribution of Dice coefficients for all four count-levels without the perturbations are shown in grey boxplots, whereas the Dice distribution with the affine perturbation of the MR images are shown in black boxplots. It can be observed that the uncertainty of the registration for different count-levels is mostly driven by the count-level, with limited effect of the perturbations. Note that all the previously presented results had the perturbation of the affine transform of the NIfTI MR image switched on by default.

This would suggest that in the investigation conducted by [5], the simulated MR perturbation of the affine matrix was not the main contributor of the similarly observed registration uncertainty, but rather the different noise levels, scanner and reconstruction types in the large number of considered PET images. The finding that the noise and registration software are among the main contributors of the registration uncertainty gives much greater control of the registration precision for future studies, as it is much more difficult to control

the positioning of participants across MR and PET scanners.

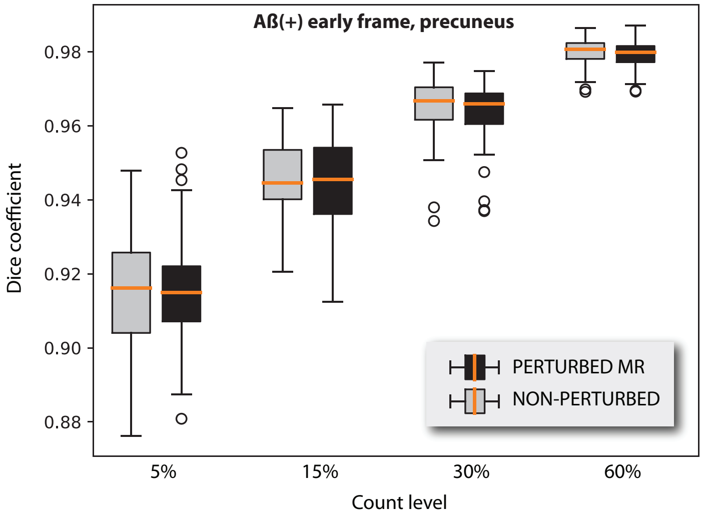

Figure 12: The effects of relative initial position of MR and PET images (randomised) on uncertainties of MR-PET registration. The effects are shown in black for the precuneus, as marked in Fig. 8. The uncertainties without perturbing the relative positions are shown in grey boxplots.

The effect of MR-PET registration uncertainty on the SUVr–the quantitative image endpoint

The distributions of the SUVr, solely due to the MR-PET registration uncertainty using the SPM software (which achieved the lowest uncertainty–see Fig. 9), for PET images with and without PVC and at four different count levels, are shown in Fig. 13. Importantly, these distributions reflect only one of a few factors affecting the final uncertainty of the SUVr statistic. That is, since the target images were kept constant, only the ROI sampling was changing due to the registration imprecision, and consequently, the effects of μ-map inaccuracy and misregistration, T1w-based image parcellation inaccuracy and the PET noise itself have not been accounted for in the distributions. The largest peak-to-peak variability for SPM12 was observed for the cingulate gyrus and was 1.297-1.343 and for non-PVC and 1.046-1.138 for PVC amyloid negative case. The understanding of the scope of the imprecision is particularly important for

longitudinal imaging, for which reducing any point statistic uncertainty leads to earlier detection of the amyloid deposition changes in the brain, whether due to deterioration or improvement as a response to treatment.

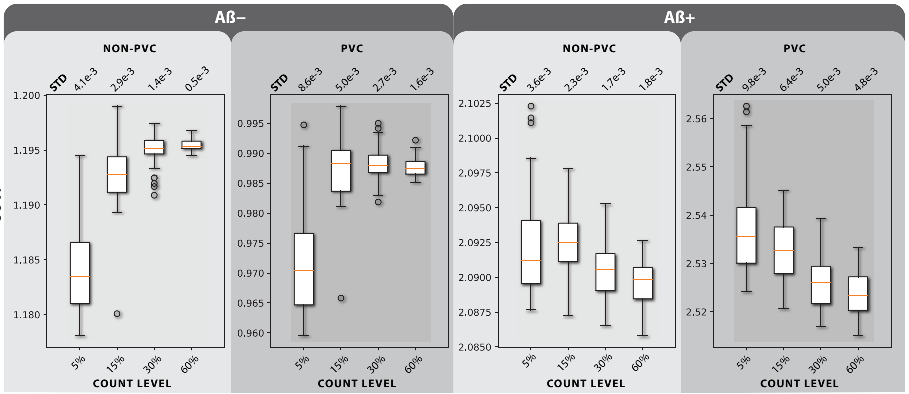

Figure 13: The effects of SPM MR-PET registration uncertainty on the precision of the SUVr for the precuneus ROI normalised to the cerebellar grey matter ROI, reported as boxplots for each count level, with and without PVC, and for negative and positive amyloid PET. Standard deviation (STD) above the boxplots.

Computational cost

With the dedicated software package *NiftyPET* for high-throughput raw data processing, it was possible to generate large numbers of independent PET scan realisations with variable noise levels and exactly the same underlying PET distribution. The performed uncertainty analysis for the two dynamic scans with two time frames, early and late, was computationally and memory intensive. It involved generating in excess of 1 TB of image data plus all the intermediate transformations. The generated PET list-mode datasets, each of around 10 GB, were calculated on the fly on the GPUs and were not stored at any point of the analysis. The biggest challenge was to maintain the large number of PET image files generated for the different aspects of the uncertainty analysis combined with four registration results for each realisation, not counting any intermediate images for calculating Dice and PET statistics.
